# Early‐life atomic‐bomb irradiation accelerates immunological aging and elevates immune‐related intracellular reactive oxygen species

**DOI:** 10.1111/acel.13940

**Published:** 2023-08-04

**Authors:** Tomonori Hayashi, Naohiro Kato, Keiko Furudoi, Ikue Hayashi, Seishi Kyoizumi, Kengo Yoshida, Yoichiro Kusunoki, Kyoji Furukawa, Misa Imaizumi, Ayumi Hida, Osamu Tanabe, Waka Ohishi

**Affiliations:** ^1^ Department of Molecular Biosciences Radiation Effects Research Foundation Hiroshima Japan; ^2^ Biosample Research Center Radiation Effects Research Foundation Hiroshima Japan; ^3^ Department of Statistics Radiation Effects Research Foundation Hiroshima Japan; ^4^ Central Research Laboratory Hiroshima University Faculty of Medicine Graduate School of Biomedical and Health Sciences Hiroshima Japan; ^5^ Biostatistics Center Kurume University Kurume Japan; ^6^ Department of Nagasaki Clinical Studies Radiation Effects Research Foundation Nagasaki Japan; ^7^ Department of Hiroshima Clinical Studies Radiation Effects Research Foundation Hiroshima Japan

**Keywords:** atomic bomb, central memory, effector memory, immunological aging, naïve, radiation, reactive oxygen species, T cell

## Abstract

Reactive oxygen species (ROS) play an important role in immune responses; however, their excessive production and accumulation increases the risk of inflammation‐related diseases. Although irradiation is known to accelerate immunological aging, the underlying mechanism is still unclear. To determine the possible involvement of ROS in this mechanism, we examined 10,023 samples obtained from 3752 atomic‐bomb survivors in Hiroshima and Nagasaki, who participated in repeated biennial examinations from 2008 to 2016, for the effects of aging and radiation exposure on intracellular ROS (H_2_O_2_ and O_2_^•−^) levels, percentages of T‐cell subsets, and the effects of radiation exposure on the relationship between cell percentages and intracellular ROS levels in T‐cell subsets. The cell percentages and intracellular ROS levels in T‐cell subsets were measured using flow cytometry, with both fluorescently labeled antibodies and the fluorescent reagents, carboxy‐DCFDA and hydroethidine. The percentages of naïve CD4^+^ and CD8^+^ T cells decreased with increasing age and radiation dose, while the intracellular O_2_^•−^ levels in central and effector memory CD8^+^ T cells increased. Additionally, when divided into three groups based on the percentages of naïve CD4^+^ T cells, intracellular O_2_^•−^ levels of central and effector memory CD8^+^ T cells were significantly elevated with the lowest radiation dose group in the naïve CD4^+^ T cells. Thus, the radiation exposure‐induced decrease in the naïve CD4^+^ T cell pool size may reflect decreased immune function, resulting in increased intracellular ROS levels in central and effector memory CD8^+^ T cells, and increased intracellular oxidative stress.

## Graphical abstract

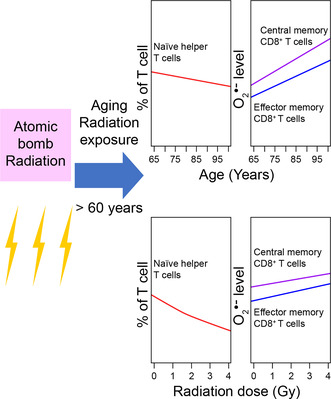

## 1 INTRODUCTION

The immune system matures by deploying number of responsive lymphocytes in the body and leaving behind memory cells after infection. However, the function of the immune system declines gradually with age, while chronic inflammation and autoimmune responses are enhanced, resulting in metabolic diseases, cardiovascular diseases, cancer, diabetes, and other attributable age‐related diseases (Bulati et al., 2017; Furman et al., 2019). Recently, a study examined the association between a number of age‐related diseases and immune markers by means of immune‐omics analysis of blood from about 1000 individuals aged 8–96 years and used deep‐learning methods to analyze the data thus obtained (Sayed et al., 2021). These age‐related functional changes in the immune system are collectively termed “immunosenescence” and are particularly marked by changes in the acquired immune system, which is characterized by antigen specificity and immune memory. However, the molecular and cellular biological bases of these changes are not well understood. Aging is a robust determinant of immune cell population composition (Huang et al., 2021; Thyagarajan et al., 2022), with aging immune systems characterized by a reduced pool of naïve (N) T cells, increased pool of terminally differentiated T cells, and increased systemic inflammation (Aiello et al., 2019).

The radiation from the atomic bomb (A‐bomb) dropped on Hiroshima and Nagasaki in 1945 has increased the risk of developing certain cancers and non‐cancer diseases, including heart disease. Long‐term epidemiological and clinical studies of A‐bomb survivors have shown that there are significantly increased risks of age‐ and immune system/inflammation‐related diseases among the A‐bomb survivors (Brenner et al., 2022; Ozasa et al., 2011; Preston et al., 2003; Shimizu et al., 1990, 1992; Yamada et al., 2004). Although there is no direct evidence that radiation exposure‐accelerated immunological aging increases the risk of certain cancers or cardiovascular diseases, it has been presumed that accelerated immunological aging due to radiation exposure is associated with increased risk of age‐related diseases (Nakachi et al., 2004). The studies on immune function are conducted using a combination of functional immunological endpoints, and have demonstrated significant radiation‐related alterations in the immune system (Akiyama, 1995; Fujiwara et al., 1994; Yamaoka et al., 2004). Importantly, many of the noted radiation effects on the immune system, such as a decrease in N CD4^+^ T cell frequency and increases in inflammatory biomarkers such as interleukin‐6 and C‐reactive protein (CRP), are similar to those associated with natural aging (Hayashi et al., 2003, 2012, 2005). These findings imply that radiation exposure accelerates immunological aging, and that such age‐related changes in the immune system may play a critical role in age‐related morbidity and mortality of A‐bomb survivors.

Reactive oxygen species (ROS) are one of the major determinants of aging, and the progressive and irreversible accumulation of ROS‐induced oxidative damage during the aging process adversely affects the molecular mechanisms of aging, thereby altering the physiological functions of the body, increasing the risk of various aging‐related diseases, and impacting life span (Harman, 1956, 2006; Kregel & Zhang, 2007). Oxidative stress occurs due to the disruption of the prooxidant–antioxidant homeostasis, resulting in the production of ROS that have deleterious effects on cell membranes, proteins, nucleic acids, and other organic compounds (Djordjević et al., 2008; Sen et al., 2010). Recently, we found that an increase in the levels of certain intracellular ROS in A‐bomb survivors might be linked to enhanced inflammatory status, including elevated serum CRP and reduced serum iron levels. These findings suggested that aging and radiation exposure increase the oxidative stress in blood cells, which is involved in accelerated immunological aging and preclinically persistent inflammation in radiation‐exposed individuals (Hayashi et al., 2021).

T cells are important host‐mediated immune cells that must respond rapidly to foreign substances and proliferate efficiently. Their growth, proliferation, and differentiation involve metabolic reprogramming in response to bioenergetic needs. Although their associated energy production inevitably generates ROS and damages cells, ROS also act as critical signaling components in T cell immunity (Peng et al., 2021). Our previous studies have assessed the effects of radiation exposure on immunological aging, but the effects of radiation exposure on the relationship between immunological senescence, particularly the decrease in N T‐cell population, and intracellular ROS have not yet been examined. In the present study, the effects of aging and radiation exposure on the relationship between the percentage of T‐cell subsets and intracellular ROS levels were examined longitudinally by measuring these parameters multiple times in the same individuals, over a period of about 7 years. We observed that aging and radiation exposure accelerated the negative association between the percentage of N T cells and intracellular ROS levels.

## 2 RESULTS

### 2.1 Characteristics of the subjects

The characteristics of the participants according to their radiation exposure status are shown in Table 1. The total number of participants was 3752, with about 63% of them being 10 years old or younger at the time of the bombings, and the average age being 9.1 years. These parameters were measured at least once between 2008 and 2016 in the participants, while they were measured at least twice during the study period in a total of 3123 participants, with an average of 2.7 measurements per participant every 2 years. In the total study population, 389 participants passed away during the study period. In terms of sex, there was no significant difference in the proportion of women between the nonexposed (<5 mGy) and exposed (≥5 mGy) groups. The numbers of participants by dose groups were 1759 for 5–<500 mGy (mean: 119 mGy, range: 5–499 mGy), 364 for 500 ≤ 1000 mGy (mean: 743 mGy, range: 503–995 mGy), and 249 for ≥1000 mGy (mean: 1709 mGy, range: 1001–3802 mGy). The age at the time of the bombings and that at the time of the examination were lower in the exposed group than in the nonexposed group. The proportion of smokers and body mass index were not significantly different between the nonexposed and exposed groups.

**TABLE 1:** Baseline characteristics of the participants.

Variable		Total	Nonexposed	Exposed
			(<5 mGy)	(≥5 mGy)
Participants	Number	3752	1380	2372
Sex	Male (%)	38.5	36.7	39.6
	Female (%)	61.5	63.3	60.4
Examinations	Number	10,023	3511	6512
Number of examinations per participants	Mean (SD)	2.7 (1.1)	2.5 (1.0)	2.7 (1.1)
Age at the time of bombing (years)	Mean (SD)	9.1 (7.3)	9.9 (7.2)	8.6 (7.3)
Age at the time of examination (years)	Mean (SD)	74.7 (7.3)	75.8 (7.2)	74.0 (7.4)
Smoking habit	Never/past (%)	91.5	91.9	91.3
	Light (%)	3.6	4.1	3.4
	Moderate (%)	3.9	3.3	4.2
	Heavy (%)	1.0	0.7	1.1
Drinking habit	Never (%)	31.8	33.4	30.8
	Past (%)	40.4	38.9	41.3
	Current (%)	27.8	27.7	27.9
Body mass index (kg/m^2^)	Mean (SD)	22.9 (3.4)	22.8 (3.4)	22.9 (3.4)
Iron (μg/dL)	Mean (SD)	97.3 (35.5)	95.4 (34.5)	98.4 (36.1)
Ferritin (ng/mL)	Mean (SD)	112.4 (124.3)	114.1 (146.2)	111.4 (109.6)
CRP (mg/dL)	Mean (SD)	0.2 (0.4)	0.2 (0.4)	0.2 (0.4)

*Note*: Smoking habit: never/past, light, moderate, and heavy smokers, depending on the number of cigarettes smoked per day: 0, 1–14, 15–24, and ≥25, respectively.

Abbreviation: SD, standard deviation.

### 2.2 Changes in intracellular ROS levels with sex, age, and radiation dose

**FIGURE 1:**
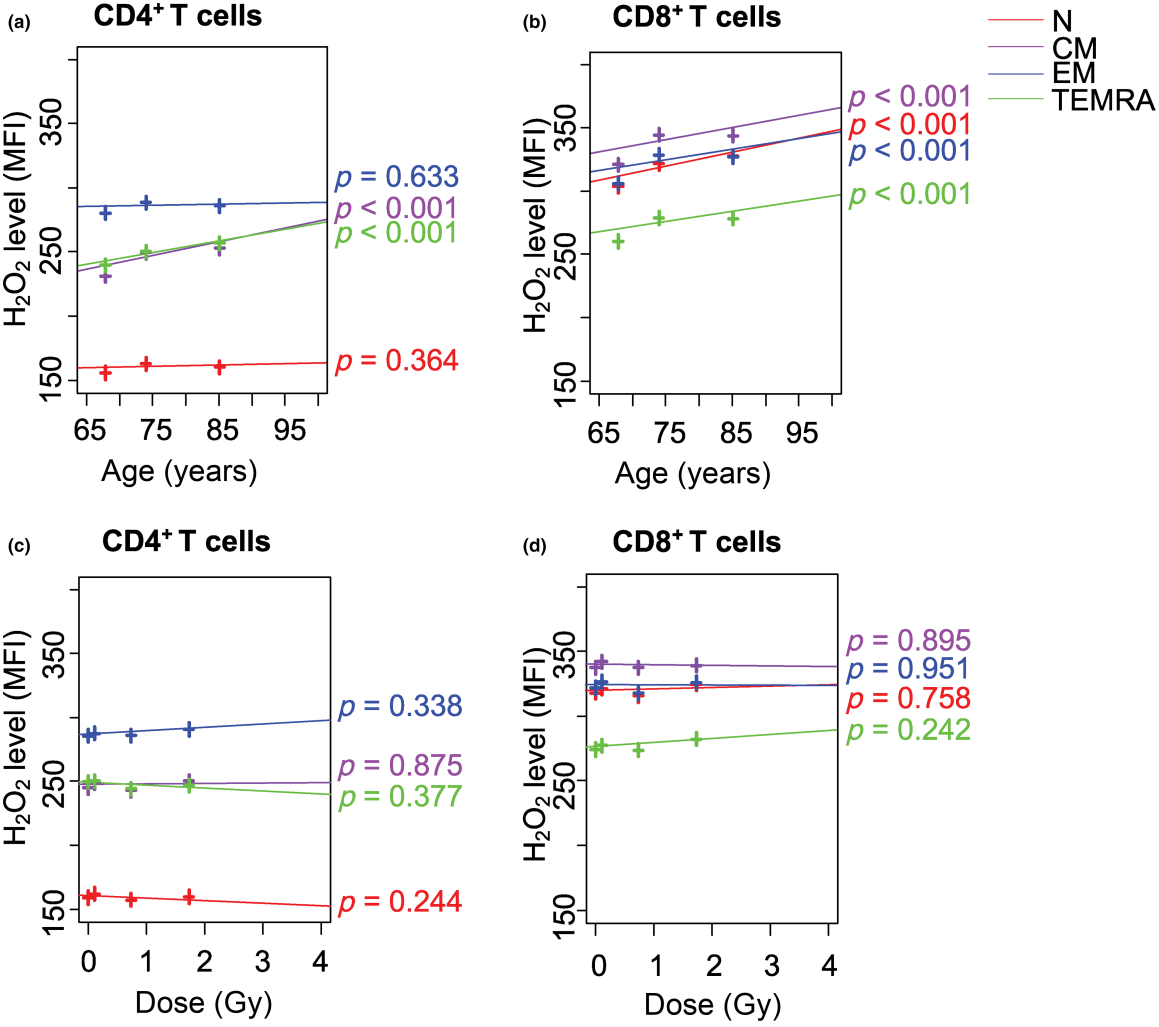
Effects of age and radiation dose on intracellular H_2_O_2_ (DCF) levels in T‐cell subsets. H_2_O_2_ intensity in N, CM, EM, TEMRA CD4^+^ T cells versus (a) age and (c) radiation dose. H_2_O_2_ intensity in N, CM, EM, TEMRA CD8^+^ T cells versus (b) age and (d) radiation dose. N, naïve (red); CM, central memory (purple); EM, effector memory (blue); TEMRA, terminal memory reexpressing CD45RA (green); DCF, dichlorofluorescein. MFI: mean of fluorescence intensity.

**FIGURE 2:**
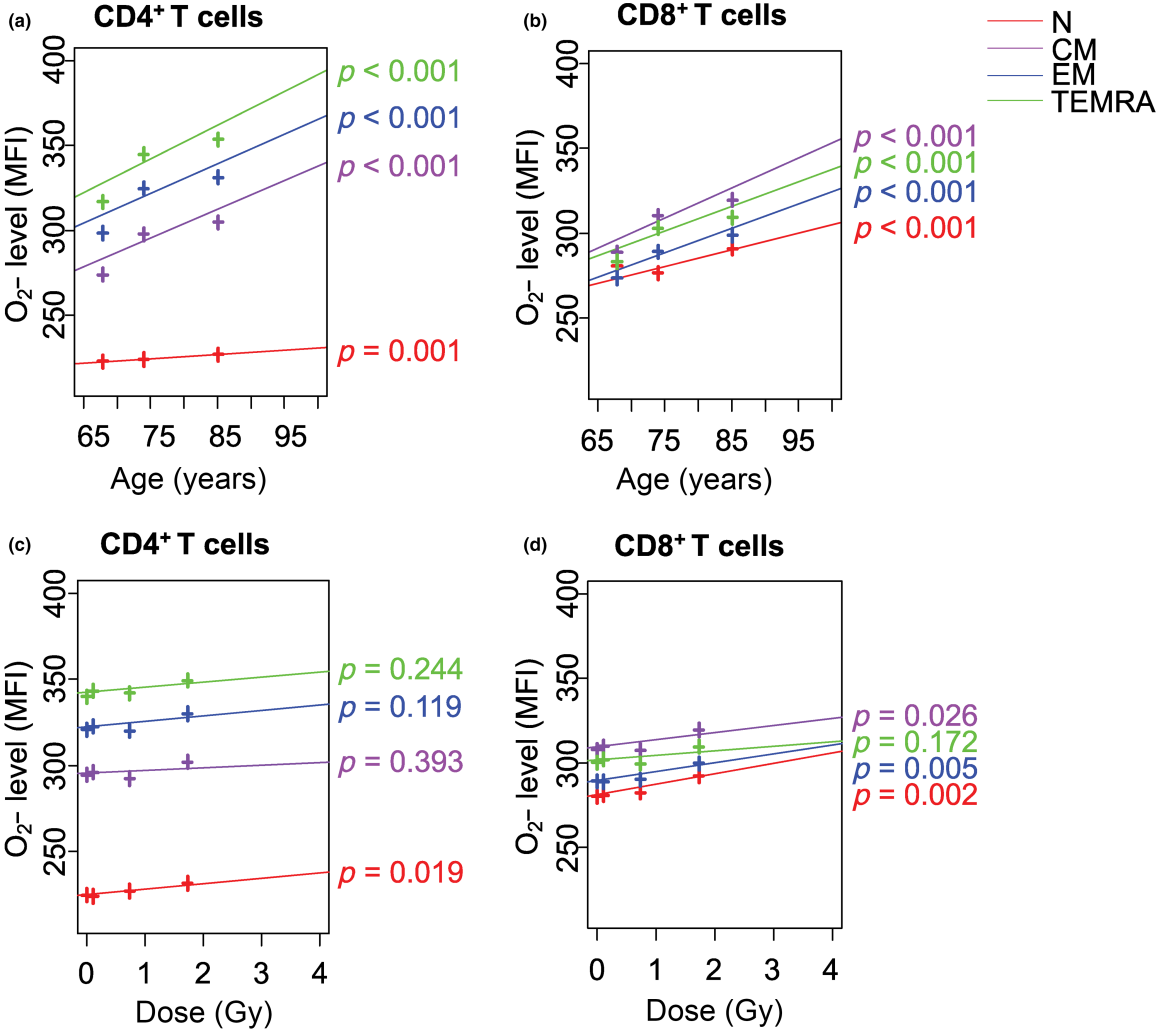
Effects of age and radiation dose on intracellular O_2_^•−^ (HE) levels in T‐cell subsets. O_2_^•−^ intensity in N, CM, EM, TEMRA CD4^+^ T cells versus (a) age and (c) radiation dose. O_2_^•−^ intensity in N, CM, EM, TEMRA CD8^+^ T cells versus (b) age and (d) radiation dose. N, naïve (red); CM, central memory (purple); EM, effector memory (blue); TEMRA, terminal memory reexpressing CD45RA (green); HE, hydroethidine. MFI: mean of fluorescence intensity.

Intracellular H_2_O_2_ levels of all T‐cell subsets, including N, central memory (CM), effector memory (EM), terminal effector memory reexpressing CD45RA (TEMRA) CD4^+^ and CD8^+^ T cells, were higher in females than in males (*p* < 0.001 for all, Table S1), while those of CM and TEMRA CD4^+^ T cells, as well as, all subsets of CD8^+^ T cells increased with age (*p* < 0.001 for all, Figure 1a,b and Table S1). However, there was no significant change in intracellular H_2_O_2_ levels with increasing radiation dose (Figure 1c,d and Table S1). On the other hand, the intracellular O_2_^•−^ levels were lower in women than in men for all CD4^+^ and CD8^+^ T‐cell subsets (*p* < 0.001 for all, Table S1). This gender‐specific difference may be due to sex hormone differences, genetic factors, and differences in immune response, but further studies are needed to confirm the reasons for this difference. Intracellular O_2_^•−^ levels of all T‐cell subsets also increased with age (*p ≤* 0.001, Figure 2a,b, and Table S1). Of note, intracellular O_2_^•−^ levels of N CD4^+^ T cells, and N, CM, and EM CD8^+^ T cells increased with radiation dose (*p* = 0.002 to *p* = 0.026; Figure 2c,d and Table S1).

### 2.3 Changes in the percentages of T‐cell subsets with sex, age, and radiation dose

**FIGURE 3:**
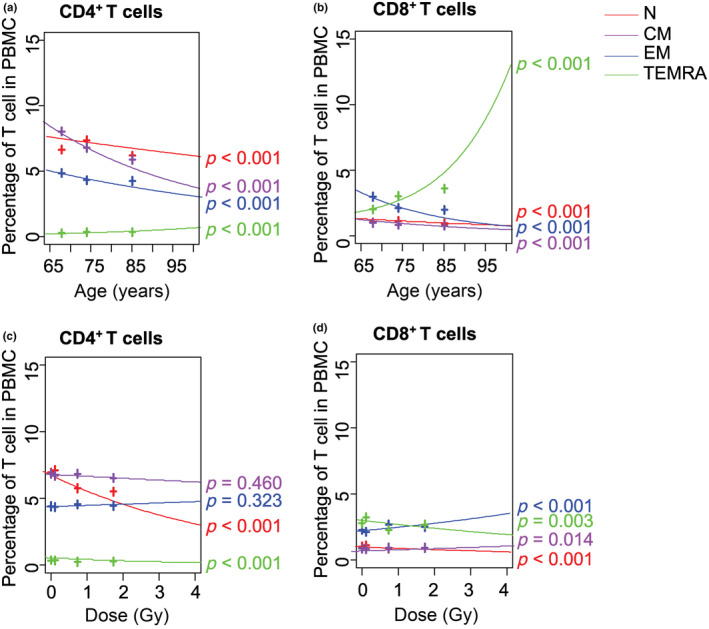
Effects of age and radiation dose on percentages of T‐cell subsets. (a) Percentages of N, CM, EM, TEMRA CD4^+^ T cells in PBMCs versus (a) age and (c) radiation dose. Percentages of N, CM, EM, TEMRA CD8^+^ T cells in PBMCs versus. (b) age and (d) radiation dose. N, naïve (red); CM, central memory (purple); EM, effector memory (blue); TEMRA, terminal memory reexpressing CD45RA (green); PBMCs, peripheral blood mononuclear cells.

The effects of sex, age, and radiation dose on the percentages of T‐cell subsets were analyzed: the percentages of N, CM, and EM CD4^+^ and CD8^+^ T cells were higher in women than in men (*p* < 0.001 for all, Table S2), while the percentage of TEMRA CD8^+^ T cells only was lower in women than in men (*p* = 0.023). The percentages of N, CM, and EM of both CD4^+^ and CD8^+^ T cells decreased with age (*p* < 0.001, Figure 3a,b and Table S2), while the percentages of TEMRA CD4^+^ and CD8^+^ T cells increased with age (*p* < 0.001 both).

The percentages of N and TEMRA CD4^+^ and CD8^+^ T cells decreased with increasing radiation dose (*p* < 0.001 to *p* = 0.003, Figure 3c,d and Table S2), while the percentages of CM and EM CD8^+^ T cells increased with increasing radiation dose (*p* = 0.014 and *p* < 0.001, respectively). The effects of sex, age, and radiation dose on the cell count of T‐cell subsets in the blood were similar (Table S3).

Although the results upon evaluating the interaction of radiation dose and age on the decrease in the percentage of N CD4^+^ and CD8^+^ T cells were not statistically significant, there was a trend suggesting that the decrease in percentage with age may be accelerated by an increase in radiation dose. The possibility that radiation may accelerate immunological aging should still be considered, even if statistical significance is not attained. These data, which are the results of a longitudinal study, with the same subjects measured multiple times, suggest that an interaction between age and radiation may exist, and may be an important part of understanding the mechanisms of immunological aging and contributing to the development of future preventive measures and treatments. These results suggested that the decrease in the proportion of N CD4^+^ and CD8^+^ T cells with increasing age may be accelerated by increasing radiation dose. We believe that these N T cell populations are the most appropriate parameters reflecting aging and radiation effects on the immune system, since we have reported in several papers over the last two decades that this percentage of N T cells decreases with increasing age and radiation (Akiyama, 1995; Hayashi et al., 2012; Yamaoka et al., 2004).

### 2.4 Relationship between percentages of N T cells and the radiation‐dependent increase of O_2_ ^•−^ levels in T‐cell subsets

**FIGURE 4:**
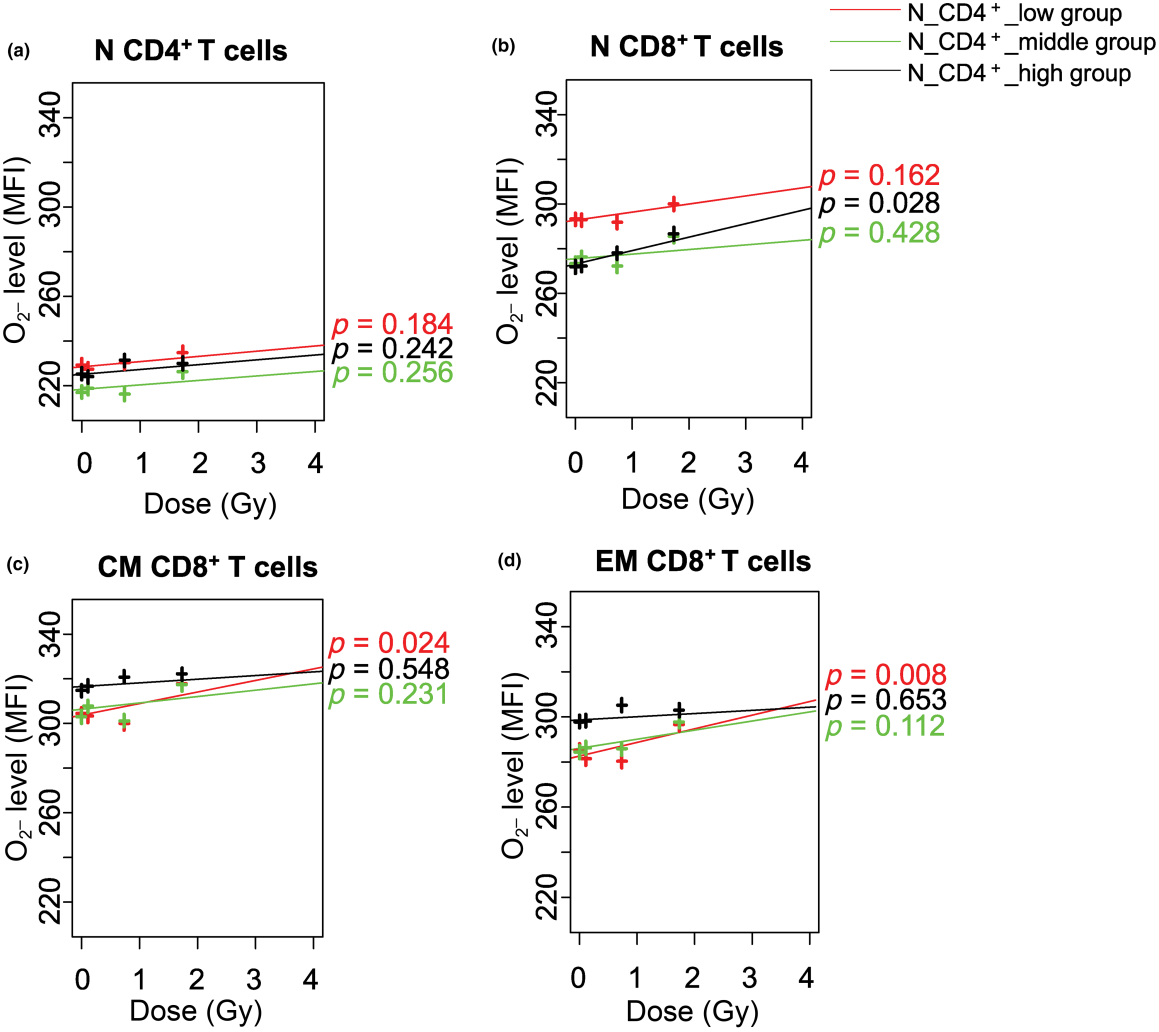
Effects of radiation dose on intracellular O_2_^•−^ levels in N, CM, and EM CD4^+^ and CD8^+^ T cells, by three groups of N CD4^+^ or CD8^+^ T‐cell percentages. (a) N CD4^+^, (b) N CD8^+^, (c) CM CD8^+^, and (d) EM CD8^+^ T cells by three groups of N CD4^+^ T‐cell percentages. Groups with low, medium, and high percentage of N CD4^+^ or CD8^+^ T cells have been indicated using red, green, and black lines, respectively. N, naïve; CM, central memory; EM, effector memory; HE, hydroethidine; CD, cluster of differentiation. MFI: mean of fluorescence intensity.

Since the percentage of N CD4^+^ or CD8^+^ T cells decreased with increasing age and radiation dose, we presumed that the decrease of N CD4^+^ or CD8^+^ T cell population may be associated with a dose‐dependent increase of O_2_^•−^ levels in the T cell subsets. Thus, we analyzed the relationship between radiation dose and O_2_^•−^ levels in N CD4^+^ T cells, and N, CM, and EM CD8^+^ T cells in groups according to the percentage of N CD4^+^ or CD8^+^ T cells. When divided into three groups according to the percentage of N CD4^+^ T cells of the corresponding examination (low group: mean = 3.20%, range = 0.03%–5.38%, *n* = 1805 subjects, radiation dose: mean 0.28 Gy, range 0.00–3.80 Gy; middle group: mean = 7.55%, range = 5.39%–10.03%, *n* = 1913 subjects, radiation dose: mean 0.22, range 0.00–3.79 Gy; high group: mean = 14.73%, range = 10.04%–40.17%, *n* = 1564 subjects, radiation dose: mean 0.18 Gy, range 0.00–3.50 Gy), we observed no significant changes in O_2_^•−^ levels in N CD4^+^ T cells with increasing radiation dose in these three groups (Figure 4a). On the other hand, O_2_^•−^ levels in N CD8^+^ T cells were higher in the low group of N CD4^+^ T cell percentages than in the other groups, and a trend of increase with increasing radiation dose was observed, but it was not significant (Figure 4b). However, at 0 Gy, the high group of N CD4^+^ T cell percentages had lower O_2_^•−^ levels in N CD8^+^ T cells than the other groups, but these levels increased significantly with increasing radiation dose (*p* = 0.028, Figure 4b). At 0 Gy, the O_2_^•−^ levels in CM and EM CD8^+^ T cells were also lower in the low group of N CD4^+^ T cell percentages than in the other groups, but increased significantly with increasing radiation dose (*p* = 0.024 and *p* = 0.008, respectively, Figure 4c,d). No significant changes of O_2_^•−^ levels in these CD8^+^ T cells with increasing radiation dose were observed in the other groups. This may imply that a decrease in N CD4^+^ T cell population is responsible in some way for the increase in O_2_^•−^ levels in CM and EM CD8^+^ T cells with increasing radiation dose.

We also conducted similar analyses by dividing the N CD8^+^ T cells into three groups according to their percentages. However, the difference in the percentages of N CD8^+^ T cells in the three groups was too small (low group: mean = 0.51%; middle group: mean = 1.21%; high group: mean = 2.77%), and no similar results were obtained (data not shown). These data demonstrated that radiation‐induced increases in ROS production were most evident in the individuals with a decreased N CD4^+^ T‐cell population.

## 3 DISCUSSION

Previous studies on A‐bomb survivors have reported that the percentages of immune cells, especially N CD4^+^ and CD8^+^ T cells, decrease with increasing radiation dose (Yamaoka et al., 2004). The present study investigated percentages of T‐cell subsets and the intracellular ROS (H_2_O_2_ and O_2_^•−^) levels in them, and revealed that when divided into three groups according to the percentages of naïve CD4^+^ T cells, the intracellular O_2_^•−^ levels of CM and EM CD8^+^ T cells increased significantly with increasing radiation dose in the group with the lowest percentage of N CD4^+^T cells.

Our findings may indicate a tendency for memory T cells to have higher intracellular O_2_^•−^ levels in the event of new infection or cellular injury, which may result in persistent inflammation. Memory CD8^+^ T cells have been reported to survive in vivo much longer than memory CD4^+^ T cells (Homann et al., 2001). The decrease in T‐cell receptor repertoire diversity with aging is much more pronounced in the memory CD8^+^ T‐cell population than in the memory CD4^+^ T‐cell population (Wack et al., 1998). In addition, low levels of intracellular ROS promote T‐cell activation, especially by inhibiting phosphatase and enhancing nuclear factor kappa B signaling, while high levels of ROS are detrimental to cell activation (Simeoni & Bogeski, 2015). Furthermore, aging causes mitochondrial changes, increases ROS and cytokine production, affects T‐cell subset differentiation and polarity, and supports unwanted terminally differentiated memory cell populations (Crooke et al., 2019). Aging stroma cells increase the production of interleukin‐15 and ‐6, and these cytokines act synergistically to generate and maintain memory CD8^+^ T cells, which produce cytokines, including interferon‐γ and tumor necrosis factor‐α, which stimulate ROS production (Pangrazzi et al., 2017). Thus, the increase in O_2_^•−^ levels of memory CD8^+^ T cells, especially CM and EM CD8^+^ T cells, with aging, is partly due to the increase in the aging stroma cell population in the body. If radiation exposure increases aging stroma cells, such T‐cell metabolic aging may have been accelerated by radiation exposure in the A‐bomb survivors.

Of the 3752 participants in the study, 3413 were young A‐bomb survivors (exposed at less than 20 years of age) while 339 were adult A‐bomb survivors (exposed at 20 years of age or older), and the present results mainly reflect those of the young survivors. When analyzed only for adult A‐bomb survivors, results for radiation effects on O_2_^•−^ levels showed a similar tendency to those of all subjects, but the results for adult survivors were not significant, except for CM CD8^+^, due to the small sample size (data not shown). This result suggests that the radiation effects for young A‐bomb survivors and adult A‐bomb survivors had a similar tendency.

Another interesting observation in the present study is that the O_2_^•−^ levels in N CD8^+^ T cells in a group with a high percentage of N CD4^+^ T cells increased significantly with increasing radiation dose (Figure 4b). The reason for this result is not clear, but one possibility might be that a higher percentage of virtual memory (VM) CD8^+^ T cells are present among N CD8^+^ T cells in the group with a higher percentage of N CD4^+^ T cells with increasing radiation dose, which could explain why the O_2_^•−^ levels are substantially elevated with increasing radiation dose. CD45RA^+^ CD62L^+^ VM CD8^+^ T cells are present among the N CD8^+^ T‐cell population (Hussain & Quinn, 2019), and these proliferate and produce cytokines faster after T‐cell receptor stimulation than true N T cells (Chiu et al., 2013; Haluszczak et al., 2009; Lee et al., 2013). We believe that a large part of the N CD8^+^ T‐cell population observed in the present study is constituted by VM CD8^+^ T cells and that similar to CM and EM CD8^+^ T cells, intracellular O_2_^•−^ levels in VM CD8^+^ T cells are significantly elevated with increasing radiation dose. In the present results, the proportion of N CD4^+^ and CD8^+^ T cells were positively correlated (*p* < 0.001 for both, data not shown), and an individual with a large N CD4^+^ population may also have a large N CD8^+^ T‐cell population, thereby constituting a large VM CD8^+^ T cell population. There is a need for further studies to clarify this presumption.

Based on the results of this study, we hypothesize that past radiation exposure, particularly high‐dose exposure, affects T‐cell function and enhances the persistent inflammatory state, thereby increasing CM and EM T‐cell ROS levels in the blood. To test this hypothesis, we continue to investigate changes in the immune and clinical status with radiation exposure and aging, as well as, an increased risk of disease onset due to radiation exposure in A‐bomb survivors, based on interactions between intracellular ROS levels and immune and inflammatory biomarkers. We expect that these studies will provide concrete evidence for the hypothesis of “accelerated immune aging due to radiation exposure”.

Studies of radiation exposure among A‐bomb survivors have been conducted for many years, but the effects of radiation exposure are not yet fully understood. The results showed that radiation exposure 60 years ago accelerated the age‐related decrease in the percentage of N CD4^+^ T cells in the blood, and increased ROS levels in CM and EM CD8^+^ T cells, which may be responsible for the elevated tissue damage response in vivo. These findings provide an important insight into the relationship between radiation exposure and the immune system. However, the uniqueness of the study population makes it difficult to validate the results. Further studies and validation are needed to support the conclusions reached in this study. First, it is necessary to verify whether similar results can be obtained by animal experiments using mice. Second, studies of different exposure doses and durations of exposure need to be conducted to corroborate the results of this study. Comparing results for subjects with a history of radiation therapy under different exposure conditions and tracking changes in intracellular ROS over time after exposure are important to increase the reliability of conclusions. Additionally, in relation to the results obtained in this study and the relationship to disease development, it is necessary to clarify more specific mechanisms of how the effects of radiation exposure on immunity are related to the development of aging‐related diseases and cancer. The study suggested a decrease in naïve T cells in the blood and an increase in ROS levels, but it is still unclear how these changes are involved in the development of specific diseases. Furthermore, elucidation of the mechanisms at the cellular and molecular levels is necessary to elucidate the relationship between radiation exposure and immune response. It is important to investigate in detail how changes in gene expression in immune cells, fluctuations in signaling pathways, and regulation of cytokines and immune checkpoints are affected by radiation exposure. Moreover, long‐term follow‐up of patients and workers exposed to radiation after radiotherapy or radiation accidents is also needed. This will reveal the actual effects of radiation exposure on the human immune response, and the risk of developing diseases that may result from exposure. Overall, it can be concluded that studies on the relationship between radiation exposure and immune response are still in progress and have not been fully validated. The effects of radiation exposure on immunity and its impact on health will be clarified in more detail through future research.

In conclusion, we found that radiation exposure 60 years ago accelerated a percentage decrease of N CD4^+^ T cells in the blood with aging and increased the ROS levels in CM and EM CD8^+^ T cells, which may be responsible for the elevated tissue damage response in vivo. It is expected that the results obtained here will be used to benefit studies of not only A‐bomb radiation exposure, but also the late effects of radiation exposure on immunity, such as radiotherapy and possible future nuclear accidents, as well as, the development of cancer and non‐cancer aging‐related diseases that may occur as a result of radiation exposure.

## 4 MATERIALS AND METHODS

### 4.1 Study populations

Adult Health Study is a clinical program that aims to investigate the late effects of radiation exposure on A‐bomb survivors through biennial health examinations. The study subjects were Adult Health Study participants of Hiroshima and Nagasaki, who visited the RERF for clinical health examination from 2008 to 2016, and gave peripheral blood samples for research use with written informed consent. The radiation exposure dose for each individual was estimated using bone marrow doses calculated using the dosimetry system DS02 (Cullings et al., 2006, 2014). Based on the medical history of these subjects, the absence of additional radiation exposure, radiotherapy, was verified in each of these subjects. Adult Health Study participants who had cancer or inflammation‐associated diseases (e.g., cold, chronic bronchitis, or rheumatic disease), for which elevated ROS levels have been reported, were excluded. A total of 3752 subjects were selected from the participants; 1380 subjects from the nonexposed group (<5 mGy) and 2372 subjects from the exposed group (≥5 mGy). Blood samples were collected at the time of health examination longitudinally and applied to the T‐cell subset analysis and intracellular ROS assay system within the same day. This study was reviewed and approved by the Research Protocol Review Committee and RERF Human Investigation Committee (the committee that functioned as the institutional review board at the time).

### 4.2 Measurements of T‐cell subset percentages and intracellular ROS levels

An ROS assay system for measuring the ROS levels in T‐cell subsets was conducted as per previously reported methods (Hayashi et al., 2021). Whole blood (0.5 mL) was collected into sodium heparin tubes, lysed with Dako erythrocyte‐lysing reagent (Agilent), incubated with antibody combinations against CD3, CD4, CD8, CD45RA, and CD62L (BioLegend), for 30 min on ice, and washed with phosphate‐buffered saline. The antibody‐stained cells were applied to the intracellular ROS assay system. 5‐(and‐6)‐Chloromethyl‐2′,7′‐dichlorodihydrofluorescein diacetate (CM‐DCFH‐DA, Thermo Fisher Scientific), a cell‐permeable nonfluorescent dye sensitive to intracellular redox changes, was used as the probe to identify H_2_O_2_. When CM‐DCFH‐DA enters the cell, it is cleaved by cytoplasmic esterases into CM‐DCF, which in the presence of H_2_O_2_ can be oxidized by peroxidases into the fluorescent molecule dichlorofluorescein (DCF) (Brubacher & Bols, 2001). The fluorescence signal emanating from this probe can be easily measured using flow cytometry, upon excitation of the dye by the 488 nm wavelength laser that is typically present in these instruments. CM‐DCFH‐DA has good specificity for H_2_O_2_, as the fluorescence of DCF appears to be mediated mainly by H_2_O_2_ (Myhre et al., 2003). Hydroethidine (HE, Thermo Fisher Scientific) is another probe for measuring ROS levels, which is oxidized to the fluorescent molecule ethidium bromide by O_2_^•−^ (Rothe & Valet, 1990), and can be excited using the same aforementioned 488 nm wavelength laser. CM‐DCFH‐DA (100 nM) or HE (100 nM) was added to the antibody‐stained cell suspension and incubated for 15 min at 37°C. At this point, green (filter 515–545 nm) or red (filter 670 LP) fluorescence was defined as the constitutively generated H_2_O_2_ or O_2_^•−^ level, respectively. The stained cells were read on a CyAn™ flow cytometer (Beckman Coulter, Orange County, CA, USA), with approximately 20,000 events being acquired from each preparation. The ROS levels of the CD4^+^ (helper) and CD8^+^ (cytotoxic) T‐cell (CD3^+^) subsets were analyzed: N (CD45RA^+^ CD62L^+^), CM (CD45RA^−^ CD62L^+^), EM (CD45RA^−^ CD62L^−^), and TEMRA (CD45RA^+^ CD62L^−^) T cells, according to CD3, CD45RA, and CD62L expression. The geometric mean values of the DCF or HE intensities in each cell subset were considered as the ROS levels. Antibody isotype controls were used to set negative staining criteria. The Jurkat cell line was used as a daily reference measurement with which each subject measurement was corrected. The percentage and cell count of T‐cell subsets were calculated as a percentage per peripheral blood mononuclear cells and cell count per total leukocyte count, using a modification of a previously published method (Yamaoka et al., 2004). All methods were performed in accordance with the relevant guidelines and regulations. Data were analyzed using FlowJo software (Becton Dickinson).

### 4.3 Measurements of serum iron, ferritin, and CRP levels

Serum iron and ferritin levels were measured with an autoanalyzer (Hitachi 7180) using 2‐nitroso‐5‐[N‐n‐propyl‐N‐(3‐sulfopropyl)amino]phenol (nitroso‐PSAP) and colloidal gold immunoassay (Ohishi et al., 2008), respectively. Serum CRP levels were measured using a CRP‐latex kit (Nissui Pharmaceutical Co. Ltd) (Hayashi et al., 2005). All measurements were performed for each subject using serum samples collected and stored at the same time.

### 4.4 Statistical analysis

The primary objective of the main statistical analysis was to evaluate the associations between intracellular ROS levels in T‐cell subsets, and percentage of T cell subsets, age (at the time of examination), and radiation exposure in the study population of A‐bomb survivors. Eight outcome variables (N CD4^+^, CM CD4^+^, EM CD4^+^, TEMRA CD4^+^, N CD8^+^, CM CD8^+^, EM CD8^+^, and TEMRA CD8^+^ T cells) were selected to examine the intracellular ROS levels in T‐cell subsets and percentage of T cell subsets in A‐bomb survivors. In order to analyze the longitudinal data measured multiple times for each individual, random intercept models were used to examine the effects of sex, age, and radiation dose on each of the eight outcome variables of ROS levels and percentage of T‐cell subsets, as well as the associations among them. In addition, as described in a previous paper (Hayashi et al., 2021), potential confounding factors affecting intracellular ROS levels and percentage of T‐cell subsets, including sex, city (Hiroshima or Nagasaki), age at the time of examination, smoking status (never/past, light, moderate, and heavy, depending on the number of cigarettes smoked per day: 0, 1–14, 15–24, and ≥25, respectively), drinking status (nondrinker, past drinker, and current drinker), body mass index (kg/m^2^: <18.5, 18.5–24.9, and ≥25), time of blood sampling (clock time), and serum iron, ferritin, and CRP levels were adjusted for. We performed logit transformation for percentages of T‐cell subsets and logarithm base 10 transformation for cell counts of T‐cell subsets because they were not normally distributed. The effect of radiation was evaluated by including the dose variable as a continuous variable. The number of cigarettes per day and body mass index were based on data obtained from the subjects at each health examination. Drinking status was classified based on past drinking status and data obtained from the subject at each health examination. Serum iron, ferritin, and CRP levels at each health examination were considered as independent variables, and ferritin and CRP were analyzed after transformation to logarithmic values at base 10. We also performed separate analyses for the three groups divided by the percentage of N CD4^+^ T cells. For the above analyses, we also performed separate analyses using categorical age at the time of examination (<70 years, 70–79 years, and ≥80 years) and radiation exposure (0–4.9 mGy, 5–500 mGy, 500–1000 mGy, or ≥1000 mGy) to check model fitting. The Wald test for the null hypothesis of no effect was used to evaluate the association with each variable. An evaluated effect was considered significant if the associated *p* value for the null hypothesis was <0.05. All statistical analyses were performed using R software, version 4.0.1 (R Foundation for Statistical Computing).

## AUTHOR CONTRIBUTIONS

T.H., study conception and design; T.H., Ke.F., M.I., A.H., and W.O., data acquisition; T.H., N.K., Ke.F., S.K., Ky.F., and O.T., data analysis and interpretation; T.H., N.K., I.H., S.K., K.Y., Y.K., M.I., A.H., O.T., and W.O., manuscript drafting or critical revision for important intellectual content; T.H., study supervision. All authors approved the final version of the manuscript for publication.

## FUNDING INFORMATION

This study was based on RERF Research Protocols #2–75, #1–93, and #3–07, and was supported in part by JSPS KAKENHI Grant Numbers JP15H04791 and JP24390162. The funding bodies did not participate in the study design; or in the collection, analysis, and interpretation of data; or in the writing of the report. The views of the authors do not necessarily reflect those of the two governments.

## CONFLICT OF INTEREST STATEMENT

None reported.

## Supporting information


Appendix S1

## Data Availability

All data generated and/or analyzed during this study are included in this article, and the data that support the results of this study are available from the corresponding author upon reasonable request. The data used to generate all the main figures are available in the Appendix S1.
