# Supplementary material for: Early‐life atomic‐bomb irradiation accelerates immunological aging and elevates immune‐related intracellular reactive oxygen species
Source: Aging Cell. 2023 Aug 4;22(10):e13940. doi: 10.1111/acel.13940 (PMC10577552; doi:10.1111/acel.13940)
Supplement: Supplementary file 1 — Appendix S1 [file ACEL-22-e13940-s001.docx]

**Supplementary data**

**Table S1 Multivariate linear regression analysis of intracellular reactive oxygen species levels in blood cells and T cells**

| Cell type | Female/Male | | | Age (10 years) | | | Dose (1 Gy) | | |
| --- | --- | --- | --- | --- | --- | --- | --- | --- | --- |
|  | Estimate^a^ | *p*-value | 95% CI | Estimate^b^ | *p*-value | 95% CI | Estimate^c^ | *p*-value | 95% CI |
| **DCF (H_2_O_2_)** |  |  |  |  |  |  |  |  |  |
| N CD4^+^ T cells | 11.57 | <0.001 | (8.06, 15.08) | 0.96 | 0.364 | (–1.11, 3.02) | –1.72 | 0.244 | (–4.6, 1.17) |
| CM CD4^+^ T cells | 14.81 | <0.001 | (9.54, 20.06) | 10.72 | <0.001 | (7.62, 13.83) | 0.34 | 0.875 | (–3.96, 4.65) |
| EM CD4^+^ T cells | 14.77 | <0.001 | (9.02, 20.52) | 0.83 | 0.633 | (–2.57, 4.23) | 2.30 | 0.338 | (–2.4, 6.99) |
| TEMRA CD4^+^ T cells | 11.31 | <0.001 | (5.91, 16.71) | 9.07 | <0.001 | (5.87, 12.26) | –1.99 | 0.377 | (–6.4, 2.43) |
| N CD8^+^ T cells | 23.85 | <0.001 | (17.05, 30.64) | 10.88 | <0.001 | (6.88, 14.89) | 0.87 | 0.758 | (–4.69, 6.44) |
| CM CD8^+^ T cells | 25.69 | <0.001 | (18.72, 32.68) | 9.64 | <0.001 | (5.51, 13.75) | –0.39 | 0.895 | (–6.1, 5.33) |
| EM CD8^+^ T cells | 17.77 | <0.001 | (11.32, 24.22) | 8.58 | <0.001 | (4.78, 12.38) | –0.17 | 0.951 | (–5.46, 5.13) |
| TEMRA CD8^+^ T cells | 14.06 | <0.001 | (8.66, 19.46) | 8.10 | <0.001 | (4.94, 11.27) | 2.66 | 0.242 | (–1.8, 7.13) |
| **HE (O_2_^•−^)** |  |  |  |  |  |  |  |  |  |
| N CD4^+^ T cells | –11.83 | <0.001 | (–14.59, –9.07) | 2.62 | 0.001 | (1.01, 4.23) | 2.74 | 0.019 | (0.45, 5.02) |
| CM CD4^+^ T cells | –21.94 | <0.001 | (–25.60, –18.26) | 16.88 | <0.001 | (14.71, 19.02) | 1.32 | 0.393 | (–1.71, 4.35) |
| EM CD4^+^ T cells | –18.05 | <0.001 | (–22.25, –13.84) | 17.36 | <0.001 | (14.87, 19.82) | 2.75 | 0.119 | (–0.71, 6.21) |
| TEMRA CD4^+^ T cells | –15.15 | <0.001 | (–20.31, –9.97) | 19.84 | <0.001 | (16.77, 22.88) | 2.51 | 0.244 | (–1.71, 6.74) |
| N CD8^+^ T cells | –11.95 | <0.001 | (–16.00, –7.90) | 9.89 | <0.001 | (7.50, 12.28) | 5.35 | 0.002 | (2.05, 8.66) |
| CM CD8^+^ T cells | –24.67 | <0.001 | (–28.50, –20.84) | 17.80 | <0.001 | (15.56, 20.05) | 3.60 | 0.026 | (0.44, 6.76) |
| EM CD8^+^ T cells | –11.52 | <0.001 | (–15.43, –7.60) | 14.32 | <0.001 | (12.02, 16.62) | 4.58 | 0.005 | (1.36, 7.8) |
| TEMRA CD8^+^ T cells | –8.23 | <0.001 | (–12.31, –4.15) | 14.53 | <0.001 | (12.15, 16.92) | 2.35 | 0.172 | (–1.03, 5.73) |

^a^female/male, ^b^per 10 years, ^c^per Gy. DCF, dichlorofluorescein; HE, hydroethidine

**Table S2 Multivariate linear regression analysis of percentage of T cell subsets**

| Cell type | Female/Male | | | Age (10 years) | | | Dose (1 Gy) | | |
| --- | --- | --- | --- | --- | --- | --- | --- | --- | --- |
|  | Estimate^a^ | *p*-value | 95% CI | Estimate^b^ | *p*-value | 95% CI | Estimate^c^ | *p*-value | 95% CI |
| N CD4^+^ T cells | 0.13 | <0.001 | (0.07, 0.19) | –0.05 | <0.001 | (–0.08, –0.02) | –0.19 | <0.001 | (–0.24, –0.14) |
| CM CD4^+^ T cells | 0.20 | <0.001 | (0.16, 0.23) | –0.24 | <0.001 | (–0.26, –0.22) | –0.01 | 0.460 | (–0.04, 0.02) |
| EM CD4^+^ T cells | 0.23 | <0.001 | (0.18, 0.27) | –0.13 | <0.001 | (–0.15, –0.11) | 0.02 | 0.323 | (–0.02, 0.05) |
| TEMRA CD4^+^ T cells | 0.05 | 0.232 | (–0.03, 0.13) | 0.22 | <0.001 | (0.17, 0.27) | –0.19 | <0.001 | (–0.26, –0.12) |
|  |  |  |  |  |  |  |  |  |  |
| N CD8^+^ T cells | 0.24 | <0.001 | (0.18, 0.30) | –0.06 | <0.001 | (–0.09, –0.03) | –0.12 | <0.001 | (–0.17, –0.07) |
| CM CD8^+^ T cells | 0.11 | <0.001 | (0.06, 0.17) | –0.21 | <0.001 | (–0.24, –0.18) | 0.06 | 0.014 | (0.01, 0.11) |
| EM CD8^+^ T cells | 0.14 | <0.001 | (0.09, 0.20) | –0.37 | <0.001 | (–0.40, –0.34) | 0.11 | <0.001 | (0.06, 0.16) |
| TEMRA CD8^+^ T cells | –0.08 | 0.023 | (–0.16, –0.01) | 0.60 | <0.001 | (0.56, 0.64) | –0.10 | 0.003 | (–0.17, –0.03) |

^a^female/male, ^b^per 10 years, ^c^per Gy

The estimates correspond with changes in logit of the percentage of T cell subsets

**Table S3 Multivariate linear regression analysis of cell counts of T cell subsets**

| Cell count in blood | Female/Male | | | Age (10 years) | | | Dose (1 Gy) | | |
| --- | --- | --- | --- | --- | --- | --- | --- | --- | --- |
|  | Estimate^a^ | *p*-value | 95% CI | Estimate^b^ | *p*-value | 95% CI | Estimate^c^ | *p*-value | 95% CI |
| N CD4^+^ T cells | 0.052 | <0.001 | (0.03, 0.08) | –0.039 | <0.001 | (–0.05, –0.03) | –0.077 | <0.001 | (–0.10, –0.05) |
| CM CD4^+^ T cells | 0.084 | <0.001 | (0.07, 0.10) | –0.123 | <0.001 | (–0.13, –0.11) | –0.003 | 0.697 | (–0.02, 0.01) |
| EM CD4^+^ T cells | 0.096 | <0.001 | (0.08, 0.12) | –0.075 | <0.001 | (–0.09, –0.06) | 0.008 | 0.313 | (–0.01, 0.02) |
| TEMRA CD4^+^ T cells | 0.020 | 0.274 | (–0.02, 0.06) | 0.078 | <0.001 | (0.06, 0.10) | –0.078 | <0.001 | (–0.11, –0.05) |
|  |  |  |  |  |  |  |  |  |  |
| N CD8^+^ T cells | 0.105 | <0.001 | (0.08, 0.13) | –0.042 | <0.001 | (–0.06, –0.03) | –0.049 | <0.001 | (–0.07, –0.03) |
| CM CD8^+^ T cells | 0.051 | <0.001 | (0.03, 0.08) | –0.113 | <0.001 | (–0.13, –0.10) | 0.027 | 0.016 | (0.00, 0.05) |
| EM CD8^+^ T cells | 0.066 | <0.001 | (0.04, 0.09) | –0.186 | <0.001 | (–0.20, –0.17) | 0.049 | <0.001 | (0.03, 0.07) |
| TEMRA CD8^+^ T cells | –0.033 | 0.046 | (–0.07, –0.00) | 0.228 | <0.001 | (0.21, 0.24) | –0.040 | 0.007 | (–0.07, –0.01) |

^a^female/male, ^b^per 10 years, ^c^per Gy

The estimates correspond with the log ratios of the cell counts of T cell subsets
